# Flexural Toughness Test and Inversion Research on a Thermal Conductivity Formula on Steel Fiber-Reinforced Concrete Components Post-Fire

**DOI:** 10.3390/ma15155103

**Published:** 2022-07-22

**Authors:** Huayun Li, Bingguang Chen, Kaicheng Zhu, Xiaolin Gong

**Affiliations:** 1 School of Architecture and Civil Engineering, Xihua University, Chengdu 610039, China; kocanzkc@163.com; 2 School of Emergency Management, Xihua University, Chengdu 610039, China; xiwoerlxx@163.com (B.C.); gxl798409742@163.com (X.G.)

**Keywords:** high temperature, SFRC component, ISO 834 standard, flexural toughness, thermal conductivity

## Abstract

Steel fibers are widely used because they can effectively improve the tensile, compressive and flexural properties of concrete structures. The selection of steel fiber dosage and aspect ratio at high temperature has an important impact on the flexural toughness of concrete components post-fire. In this paper, discussions are made on the simulated fire test in compliance with the ISO 834 standard to study the steel fiber-reinforced concrete (SFRC) components post-fire. The research reveals the influence of two commonly used steel fiber aspect ratios (50, 70) and steel fiber dosages (30 kg/m^3^, 40 kg/m^3^, 45 kg/m^3^) on the changes of the internal temperature field, the initial crack flexural strength and the flexural toughness of the SFRC components under a single-side fire. Moreover, combined with the four-point flexural test of the SFRC components post fire, the research also describes the damage of high temperatures to the flexural toughness of SFRC components, and suggests a calculation formula for SFRC thermal conductivity by way of the numerical inversion method. The results of this study have verified that the incorporation of steel fiber into concrete helps to reduce its internal thermal stress difference and improve the crack resistance and fire resistance of the concrete. Moreover, under high temperature conditions, the concrete component added with the steel fiber in an aspect ratio of 70 and a dosage of 45 kg/m^3^ increased their initial crack flexural strength by 56.8%, higher than that of plain concrete components, and the loss of equivalent flexural strength and flexural toughness of SFRC post fire was only 45.2% and 13.6%, respectively. The proposed calculation formula of thermal conductivity can provide a reference for a numerical simulation study of the temperature field of SFRC components in a high temperature environment.

## 1. Introduction

Concrete is an engineering composite material in which aggregates are cemented into a whole by a cementitious material. When concrete is subjected to high temperature for a long time, the concrete surface will experience explosive spalling, or even collapse, which will affect buildings, tunnel structures, and bridges, etc., causing huge economic losses and casualties [1,2,3]. In the service life of concrete structures, fire is the most frequent disaster, and the safety of concrete structures post-fire has become one of the focuses of scholars. Nowadays, scholars have carried out quite a few studies on the fire resistance of concrete structures and on the damages to concrete mechanical properties after high temperatures, and certain results have been achieved [4,5,6,7,8]. Specifically, Kodur et al. [4] believed that concrete materials were refractory because the material formed by the chemical combination of aggregate and cement in concrete was inert. However, concrete will undergo physical and chemical changes after being subjected to high temperatures, such as physical changes in pore volume, average pore size and crystal structure, and the chemical changes such as the degradation reaction of cement slurry in the heating process. The degradation rate depends on temperature, pressure and SiO_2_ content in limestone [5]. Studies by Khoury et al. [6] showed that high temperatures could cause the spalling of concrete structures and lead to them losing their load-bearing capacity above 600 °C. Xiao et al. [7] concluded that the concrete strength, elastic modulus and peak strain decreased with the increase in temperature. Anupama Krishna et al. [8] studied the influence of high temperatures on different grades of concrete, and found that the residual strength of high-strength concrete was lower than that of ordinary concrete subjected to high temperatures, and the plain concrete structure could hardly endure the long-lasting and complex high-temperature environment [9,10,11].

In recent years, fiber has become a commonly used composite material in concrete additives due to its superior fatigue resistance and durability [12,13,14,15]. There are many fiber materials that can be added to concrete, such as steel fiber, polypropylene fiber and basalt fiber. Their material properties vary. The addition of steel fibers to concrete can significantly improve the tensile, compressive, flexural, corrosion resistance and high temperature resistance of concrete [16,17,18,19,20]. The addition of polypropylene fibers can optimize the pore size distribution of concrete and improve the crack resistance of concrete [14] and adding basalt fibers to concrete can improve the permeability, carbonation resistance, acid and alkali corrosion resistance, frost resistance and high temperature resistance of the structures [15]. Zaghloul et al. [21] studied the effect of fiber volume fraction and applied stress level on the fatigue life of composites, and proposed that fiber composites maintained a good initial strength retention rate after high-frequency fatigue cycles. Serrano et al. [22] put forward by means of a fire test that adding 1% polypropylene fiber and 2% steel fiber could improve the compressive strength of concrete, polypropylene fiber concrete could reduce the tension in the concrete pores at an ambient temperature of 400 °C, thereby delaying the appearance of spalling and cracks on concrete and improving the fire resistance of concrete. Based on the three-point flexural test, Czoboly et al. [23] concluded that by adding short steel fibers in a dosage of 1% under normal temperature and fire conditions, the residual flexural strength after cracking was improved. By mixing steel fibers and polypropylene fibers into high-strength performance concrete, Yan et al. [24] proved that the compressive strength, split tensile strength and flexural strength of the mixed fiber high-strength concrete after a high temperature of 800 °C were 1.24, 4.5 and 1.61 times stronger than those of ordinary concrete, respectively. Guler et al. [25] studied the effect of adding fibers on the performance of reinforced self-compacting concrete at room temperature and high temperatures. Their study showed that the steel fiber dosage of 1% helped to improve the residual compressive strength at a high temperature, the mass loss at 800 °C was reduced by 22.2% compared to reinforced self-compacting concrete and that the steel fiber could effectively improve the residual toughness. Abdi et al. [26] conducted tensile and compressive strength tests on SFRC after heating at 100–800 °C. Their study manifested that adding 0.25% and 0.5% steel fibers to plain concrete could increase the tensile strength of ordinary concrete by an average increase of 58.48% and 80.29%, respectively, and the compressive strength increased from 20 MPa to 84 MPa. Kim et al. [27] made comparisons among steel fibers in different types (crimped type or hooked-end type) and dosages (volume fraction of 0.25%, 0.5% and 1%) after high temperatures (room temperature, 300 °C, 500 °C and 700 °C) to carry out mechanical property tests, and they showed that the tensile strength would be affected by the volume fraction of fibers and the aspect ratio. In terms of SFRC fluidity, they found that the fluidity decreased gradually with the increase in fiber content. For example, adding 0.5–1.5% of steel fibers to concrete could reduce concrete slump by 65–90 mm [28]. Under the same dosage, the fluidity of the hooked-end steel fiber was lower than that of crimped type [29]. Düğenci et al. [30] believed that as the amount of steel fibers increased, some steel fibers would be bonded, resulting in local voids in the concrete and a decrease in compressive strength. Zhang et al. [31] believed that the crimped type steel fibers were better than hooked-end steel fibers in enhancing the compressive strength, splitting strength, shear strength and flexural strength of concrete. The above studies demonstrate that adding steel fibers to concrete can improve the mechanical properties of concrete at room temperature and high temperature. However, whether the addition of steel fibers will change the internal thermal parameters of concrete is worthy of further discussion. The study of Kodur et al. [32] showed that in the range of 0–1000 °C, the influence of steel fibers on the specific heat of concrete was very small. Liu et al. [33] proved through experiments that the two-phase theoretical model of limestone and cement mortar was not suitable for studying the thermal conductivity of fiber-reinforced concrete, and thus proposed that the three-phase series-parallel model of fiber, limestone and cement mortar was better for that purpose. Based on the composite material theory, Bouziadi et al. [34] used the ANSYS numerical simulation to prove that the two-phase series model of thermal conductivity and specific heat was suitable for analyzing the thermal conductivity of SFRC. Liang et al. [35] found through the ANSYS numerical simulation that adding steel fibers could significantly improve the thermal conductivity of concrete, whereas the effect of the aspect ratio of steel fibers on the thermal conductivity of concrete was not obvious. Sargam et al. [36] added steel fibers with a volume fraction of 0.25–2% to improve the thermal conductivity of concrete, whereas adding polypropylene fibers had little effect.

The above analyses manifest that many achievements have been made in the research on the mechanical properties and thermal conductivity of fiber-reinforced concrete structures, but there is still room for improvement. The first question is how to choose fibers. Polypropylene fiber has a low melting point and poor heat resistance, which, when encountering high temperatures, will melt into pores and weaken the mechanical properties of concrete [23,24,25,37,38], making it hard to deal with long duration of fire. Both basalt fiber-reinforced concrete and SFRC have a better heat resistance, and the residual mechanical strength of SFRC subjected to high temperature is higher than that of basalt fiber reinforced concrete [39]. Therefore, upon comprehensive comparison, steel fiber was selected as the research object. Secondly, at present, most fiber concrete fire tests are heated by resistance furnaces [23,24,25,26,27,30,37,38,39]. Under the high temperature of the resistance furnace, the temperature field distribution gradients inside and outside the concrete specimens tend to be consistent, and it is impossible to achieve a single-sided fire effect. However, in real life, concrete structures are generally exposed to a single-sided fire [40,41], so the internal temperature-time variation of SFRC specimens cannot be rigorously simulated by resistance furnace heating. In addition, although there have been some studies on the thermal parameters of SFRC, they have mainly focused on theoretical analyses and laboratory tests [32,33,36]. Compared with a numerical simulation, experimental research is not cost-effective and takes a long time, so numerical simulation can be an ideal substitute for experiment under certain circumstances. However, in the literature, the research on the thermal conductivity of SFRC based on experimental results and numerical simulation is very limited [34,35], meanwhile, the direct numerical simulation may cause a certain deviation from the actual engineering situation.

Therefore, to better simulate the single-sided fire effect on the damage and thermal conductivity of SFRC specimens at high temperature, the automated furnace was adopted in our test in compliance with the ISO 834 standard heating curve [42]. The SFRC specimens in different steel fiber aspect ratios and dosages were subjected to a fire simulation for 60 min till the temperature–time curve inside the specimens was obtained, and also the influence of the steel fiber on the internal temperature transfer rate and on the change law of the concrete specimens was verified. At the same time, a four-point flexural test was carried out on both the SFRC and the plain concrete specimens to explore the damage degree of high temperature and steel fiber to the equivalent flexural strength and flexural toughness of SFRC specimens. Then ABAQUS was used along with the thermal parameters in the Eurocode to conduct a numerical simulation analysis of the transient heat of SFRC components. Based on the above, a calculation formula of thermal conductivity of SFRC was thus proposed.

## 2. Experimental Program

### 2.1. ISO 834 Standard Heating Curve

The standard heating curve is the basis for studying high temperature tests, and different high temperature tests should be heated according to the corresponding heating curve. At present, the commonly used heating curves are: ISO 834 [42] formulated by the International Organization for Standardization, ASTM E119 standard heating curve adopted by the United States and Canada [43], hydrocarbon (HC) heating curve proposed by the European Committee for Standardization [44] and the RABT-ZTV heating curve proposed by the World Road Association [45], etc.

The ISO 834 standard [42] is mainly used to evaluate the load-bearing capacity, integrity and thermal insulation of samples exposed to high temperature and high pressure conditions for beams, floor materials, ceiling materials and walls. The ASTM E119 standard [43] is applicable to masonry elements and composite elements of structural materials on buildings, but the thermocouple used in the measurement of the heating curve is enclosed in an inconel protective tube; therefore the temperature taken in the tests, especially during the first 15 min, may be lower than the temperature of the furnace. As the thermocouple used in ISO 834 standard is directly exposed in the furnace, the furnace temperature can be obtained in real time. The HC heating curve [44] is mainly used to describe the high-temperature combustion process using carbon-hydrogen compounds as the main fuel. It can simulate the heating curve generated by the high temperature after large-scale leakage caused by the collision and damage of large flammable tanks such as gasoline tank and chemical transportation tanks. The RABT-ZTV heating curve [45] is suitable for the situation where the temperature rises sharply at first, reaches its height in short time, then keeps on for a period of time before it drops to an ambient temperature. It is mainly used to simulate the high temperature conditions caused by fires within fully enclosed channels, such as city subways, expressways and railways, or by the burning of fibrous items such as wood, cloth, and clothing in building structures. The ISO 834 standard heating curve [42] can better simulate the heating mode of the concrete components mainly exposed to high temperature due to the combustion of fibrous materials.

Based on the above, the ISO 834 standard [42] was adopted in the research to simulate the fire situation for SFRC components. In order to subject the specimens to a flame similar to the actual high temperature, the test was heated by an open flame. The temperature in the test furnace and the internal temperature of the specimens were collected by K-type armored thermocouples with a monitoring temperature range of 0–1300 °C, and the electrical signals generated by the thermocouples were directly transmitted to the computer. The temperature in the test furnace changed with time, and the change rule of its standard heating curve satisfied the following functional relationship, in Formula (1).

(1) $$T=345\;\log_{10}\left(8t+1\right)+20$$

where *T* is the average furnace temperature (°C); *t* is the time (min).

### 2.2. Experimental Materials and Mix Proportion

The raw materials of the concrete in the test were water, water-reducing agent, cement, fine aggregate, coarse aggregate and steel fiber, among which the water was ordinary tap water added with 0.8% carboxylic acid superplasticizer with a water-reducing rate of 30% [46]; the cement was grade 42.5 Portland cement; the fine aggregate was ordinary river sand; the coarse aggregate 3–15 mm continuous graded crushed stone; and the steel fibers were produced in Hengshui, Hebei, China, crimped type with an equivalent diameter of 0.5 mm. The equivalent diameter was defined as that calculated when the steel fiber with a non-circular cross-section was also regarded as an equivalent with a circular cross-section.

**Figure 1:**
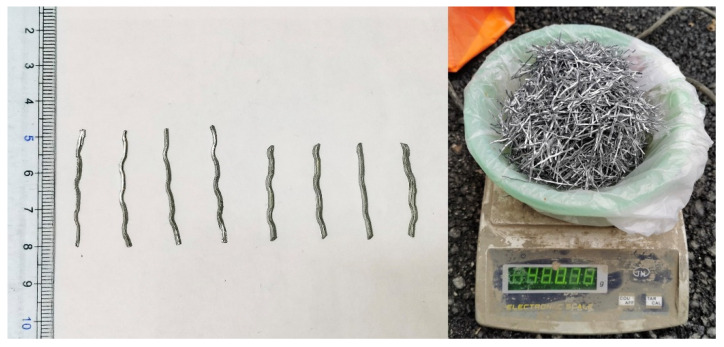
Appearance of the steel fiber.

When the length of the steel fiber is excessively long, it will cause difficulty in concrete mixing, reduce the fluidity and ultimately lead to a decrease in the strength of SFRC. Therefore, we selected the steel fibers in lengths of 25 mm and 35 mm for the test, which are commonly used in China. Their appearance and performance parameters are shown in Figure 1 and Table 1, where I is the steel fiber with a length of 25 mm and an aspect ratio of 50, and II is the steel fiber with a length of 35 mm and an aspect ratio of 70. The design grade of the base concrete strength was C35, i.e., the concrete with a compressive strength of 35 MPa measured as a cube in a side length of 150 mm after curing for 28 days at 20 ± 3 °C and humidity ≥ 90% [47]. The specific mix design is shown in Table 2.

### 2.3. Specimen Preparation

**Figure 2:**
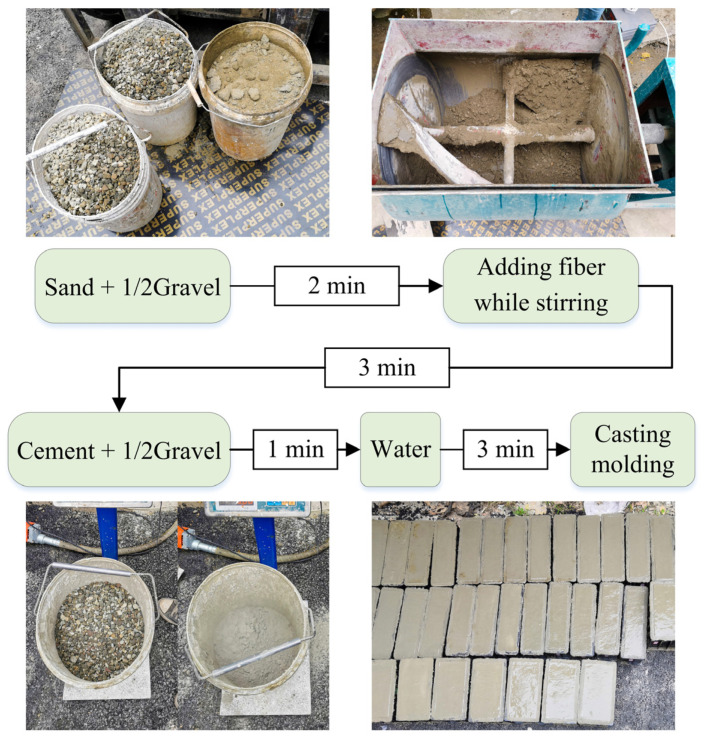
Flow chart of specimen pouring.

In order to ensure the uniformity of steel fiber stirring and enhance the bridging effect between the components of the mixture [48], the test specimens were poured by machine vibration stirring with dry mixing first and then with water [49,50]. The regulations of CECS 13:2009 [51] were strictly followed in the specific pouring process of SFRC specimens: first river sand and 600 kg/m^3^ crushed stones were added for vibrating dry mixing for 2 min, then steel fibers were added, stirring was maintained for 3 min to ensure that the steel fibers spread evenly in the concrete to prevent agglomerating, then cement and more 600 kg/m^3^ crushed stones were added for dry mixing, and finally water was added for mixing. The whole pouring process of the specimens is shown in Figure 2.

In the initial design, three steel fiber dosages of 30 kg/m^3^, 40 kg/m^3^, and 50 kg/m^3^ were selected, but as the 50 kg/m^3^ specimen was poured and stirred, the steel fibers appeared to have undergone agglomeration and had poor fluidity. The phenomenon would eventually make it hard for the fibers to be completely filled in cement mortar as holes were formed, which would first lead to the stress concentration around the holes, and then to the cracks with the increase in the stress [30]. So, the final design selected three dosages of 30 kg/m^3^, 40 kg/m^3^ and 45 kg/m^3^ and two aspect ratios of 50 and 70, for preparing 14 groups of specimens, 3 pieces in one group, and each piece in 100 × 100 × 400 mm. Moreover, the test result was decided from the average value of the flexural properties of SFRC after normal temperature and high temperature. The grouping and numbering of the specimens are shown in Table 3 and Table 4, where N and F are the normal temperature and high temperature conditions, respectively. The subscripts are fiber types and dosages: N_I-30_ is the specimen under normal temperature working condition in steel fiber Type I and a dosage of 30 kg/m^3^; N_II-45_ is the specimen under normal temperature working condition in steel fiber Type II and a dosage of 45 kg/m^3^; N_0_ is the plain concrete with 0 kg/m^3^ steel fiber.

**Figure 3:**
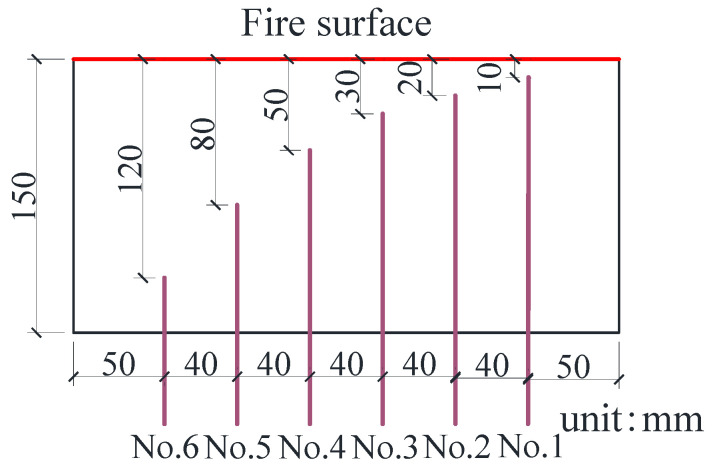
Thermocouple layout.

As thermocouples in different lengths needed to be embedded to obtain the internal temperature field of the specimens under high temperature conditions, a group of specimens in 150 × 150 × 300 mm were prepared, with one piece as one set. The grouping and numbering of these specimens are shown in Table 5, in which B is the pre-embedded thermocouple specimen. The arrangement of the pre-embedded thermocouples is shown in Figure 3.

One day after the pouring, the mold was removed and the specimens were subject to water curing for 28 days. To avoid the influence of the moisture inside the specimens on the fire test, the specimens were left standing at room temperature for 120 days to be fully air-dried before the fire test. The natural cooling down to room temperature was necessary for the flexural performance test.

### 2.4. Burning Process

**Figure 4:**
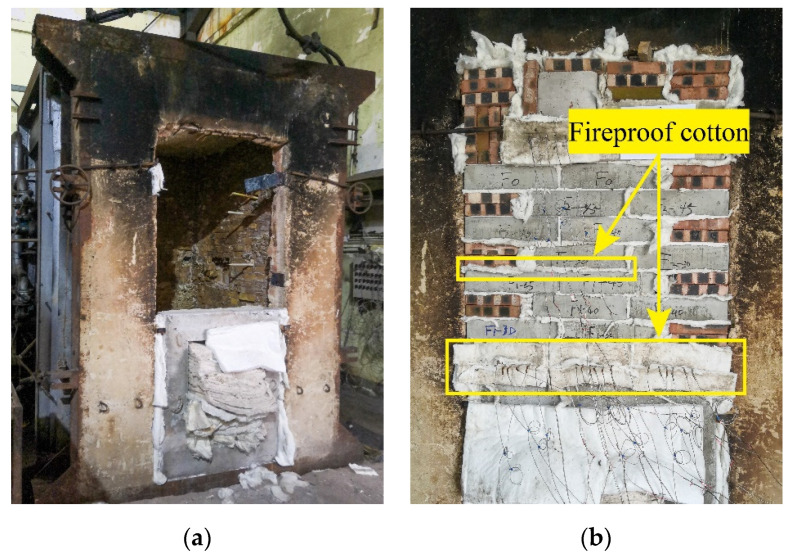
Furnace and fire layout: (**a**) vertical furnace; (**b**) masonry effect of the specimen.

This standard fire test was carried out in the Sichuan Fire Research Institute of the Ministry of Emergency Management. The furnace was composed of a furnace hearth and a vertical frame door. The furnace was 130 cm high, 110 cm wide and 100 cm deep. The specimens numbered F and B were masoned in the furnace door frame. The layout of the furnace and on-site fire is shown in Figure 4a. The masonry joints between the specimens were filled with aluminum silicate fireproof cotton, as indicated by the yellow arrow in Figure 4b, to prevent the thermal effect between the specimens during the test. The backfire surface of the specimen B was covered with fireproof cotton to prevent heat exchange with the atmosphere, reduce temperature loss and better simulate the backfire surface of the building structure at high temperature. The thermocouples were connected to the temperature control system to collect the temperature changes inside the specimens in a timely manner.

**Figure 5:**
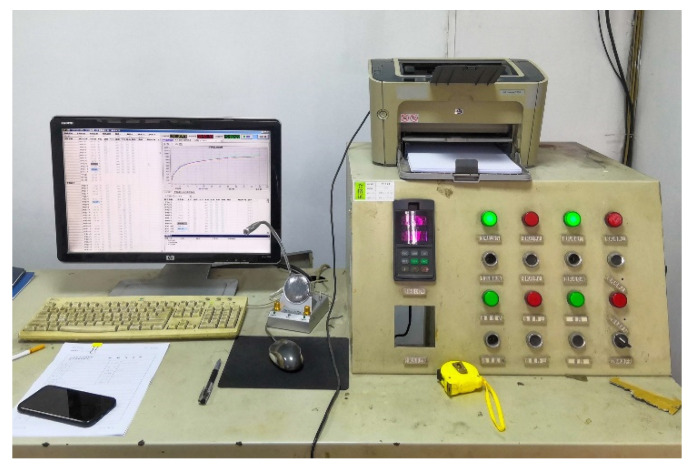
Temperature control and data collection systems.

According to a decade of statistics, about 70% of urban fires burn within 60 min [52]. With the advancement of communication technology, alarm speed and intelligent firefighting technology, the fire duration has been effectively reduced [53]. Therefore, taking into account the actual situation, the duration of the test fire was chosen as 60 min. The ambient temperature at the beginning of the test was about 15 ± 1 °C. Eight thermocouple temperature sensors were evenly arranged in the furnace. The sensors interacted timely with the temperature control system, and the real-time temperature was recorded every 1 min. When it was monitored that the temperature in the furnace was lower than the standard temperature, the fire intensity would be strengthened accordingly and vice versa. The temperature control and temperature collection systems are shown in Figure 5.

### 2.5. Flexural Toughness Test Apparatus and Procedure

The toughness index is usually used to quantitatively describe the cracked working ability, energy absorption ability and overall survivability of materials, components or structures after cracking, that is, the residual strength when large deformation occurs [54]. Among them, the flexural toughness test method can better simulate the actual stress of most engineering components, and the operation method is simple and easy to operate. It is the most popular test method for determining the energy dissipation capacity of fiber-reinforced concrete [55]. Flexural toughness characterizes the ability of a specimen to absorb energy during flexural failure and is expressed as the area of the corresponding area under the load-deflection curve.

**Figure 6:**
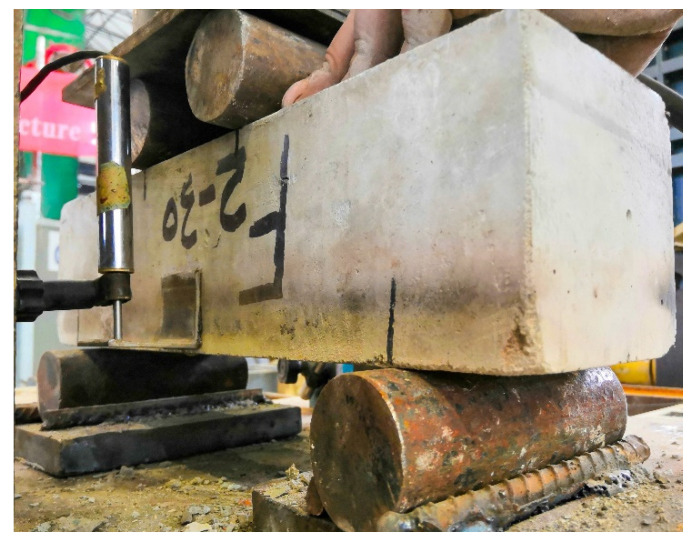
Setup chart of flexural toughness loading.

Flexural toughness tests were performed on the specimens that were naturally cooled after high temperature conditions and those under normal temperature conditions [56]. The Linear Variable Differential Transformer (LVDT) fixed at the mid-span on both sides of the specimen was used to measure the mid-span deflection. The test device is shown in Figure 6. The specific procedures were as follows: (1) start the testing machine after the specimen is stably centered, and adjust the indenter and the support to make the specimen and the indenter contact uniform; (2) install LVDT displacement gauges on both sides of the mid-span of the specimen, then carry out preloading debugging until the normal operation is obtained for the start of the formal test; (3) load the specimen continuously and uniformly at a loading rate between 0.05 and0.08 MPa/s before initial cracking, and at 0.1 mm/min afterwards, and stop the test when the mid-span deflection is greater than 3.5 mm; (4) to draw a load–deflection curve and perform a flexural toughness analysis.

## 3. Experimental Results and Discussion

### 3.1. Difference between Actual Temperature Rise and Standard Temperature Rise in the Furnace

**Figure 7:**
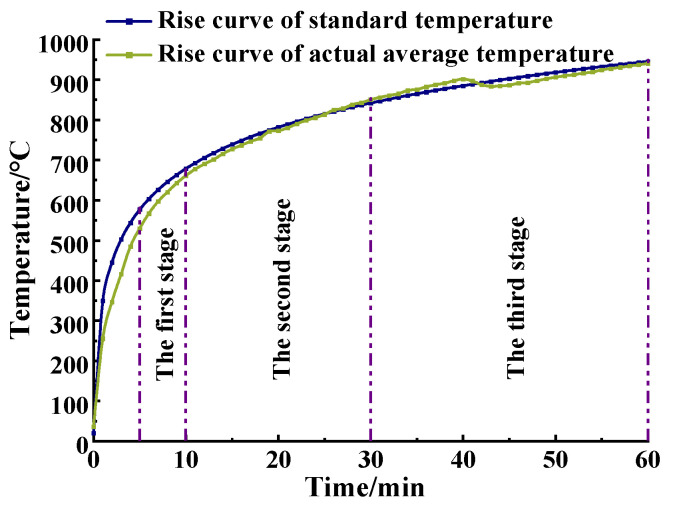
Time curve of standard/actual average temperature in the furnace.

It can be seen from Figure 7 that the growth rate of the actual temperature in the furnace changed from fast to slow in the burning process. When the burning lasted for 10 min, the air temperature in the furnace reached about 650 °C. As time went on, the temperature in the furnace kept increasing, but the growth rate gradually decreased. When the burning continued for 60 min, the temperature in the furnace was about 1000 °C. The deviation *d_e_* between the actual temperature–time curve in the furnace and the standard temperature–time curve is expressed by Formula (2) [42]. The only result within the specification limit was the deviation calculation which shows that the high temperature of the simulation scene was in line with the situation of the actual high temperature.

(2) $$d_{e}=\frac{A-A_{s}}{A_{s}}\times 100$$

where *d_e_* is the deviation, in percentage; *A* is the area under the actual average furnace time/temperature curve; *A_s_* is the area under the standard time/temperature curve; *t* is the time, in minutes. Moreover, *d_e_* should meet the following requirements:

(1) *d_e_* ≤ 15% for 5 min < *t* ≤ 10 min;
(2) *d_e_* ≤ [15 − 0.5(*t* − 10)]% for 10 min < *t* ≤ 30 min;
(3) *d_e_* ≤ [5 − 0.083(*t* − 30)]% for 30 min < *t* ≤ 60 min;
(4) *d_e_* ≤ 2.5% for *t* > 60 min.

The actual heating curve and the standard heating curve of each time period and the area enclosed by the time axis were obtained; thus, the area deviation of each section in Figure 7 was finally acquired: the first section *d_e_*_1_ = −4.53%, the second section *d_e_*_2_ = −0.82%, and the third section *d_e_*_3_ = −0.24%. The deviation calculation result was within the specification limit, indicating that the temperature control in the entire combustion heating test furnace met the requirements, the high temperature scene simulated by the test was in line with the actual high temperature situation and subsequent evaluation analysis could be carried out on the specimens under high temperature working conditions.

### 3.2. Distribution of Temperature Field Inside the Specimens

**Figure 8:**
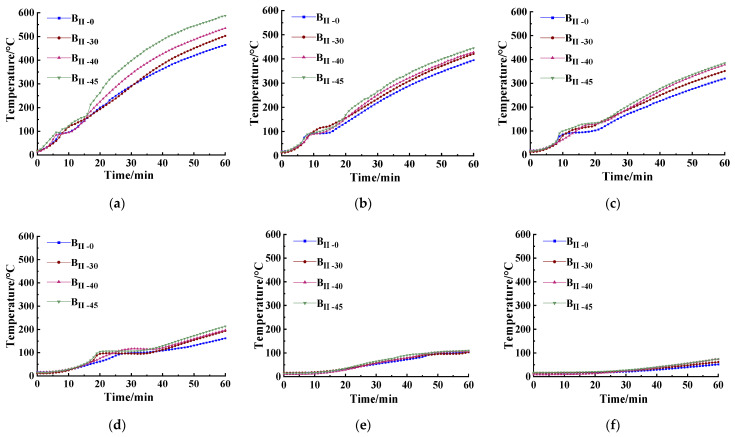
Temperature–time curves at different distances from the fire surface: (**a**) 10 mm; (**b**) 20 mm; (**c**) 30 mm; (**d**) 50 mm; (**e**) 80 mm; (**f**) 120 mm.

After ignition, the temperature in the furnace increased rapidly, and the temperature inside the specimens in four steel fiber dosages and in different distance as of 10, 20, 30, 50, 80, and 120 mm from the fire surface is shown in Figure 8. It can be seen that when the temperature inside the SFRC specimen reached 100–110 °C, it stopped increasing. A small section of obvious temperature plateau appeared in the curve. This phenomenon was mainly due to the fact that when the internal temperature of the specimen reached 100 °C, the moisture inside gradually began to evaporate, and the evaporation of water absorbed the heat from the fire surface. However, the internal temperature of the specimen continued to rise when the water inside was completely evaporated. Liu et al. [57] and others have also confirmed this.

It can be seen from Figure 8 that the internal temperatures of specimens in different steel fiber dosages all increased with the heating time, and after 110 °C the temperature curve turned roughly in a linear line. Most of the time, the temperature inside SFRC was TB_II-45_ > TB_II-40_ > TB_II-30_ > TB_II-0_. This is because the thermal conductivity of steel fibers is stronger than that of concrete, so as the amount of steel fibers increases, the temperature in the specimens increases. Given the limited burning time and the comparatively longer distance from the fire surface, the data after 110 °C were selected for analysis only to avoid the influence of the temperature platform on the specimens. The internal temperatures of the specimens shown at 80 mm and 120 mm distances from the fire surface did not exceed 110 °C in the fire test, so they were excluded from the in-depth analysis. Table 6 shows the final temperature of the specimens in different dosages and from different fire exposure distances.

Table 6 shows that in the same distance from the fire surface, the average temperature propagation speed inside SFRC became faster with the increase in the steel fiber dosages. The temperature changes of TB_II-45_, TB_II-40_ and TB_II-30_ were 23.5%, 11.3% and 1.8% higher than those of TB_II-0_, respectively. The results show that the incorporation of steel fibers into concrete can help cut down the temperature gradient inside the concrete, reduce the thermal stress difference caused by the temperature difference and inhibit the generation and development of small cracks in the specimen, thereby improving the crack resistance and fire resistance of the concrete.

### 3.3. Flexural Toughness before and after Fire

**Figure 9:**
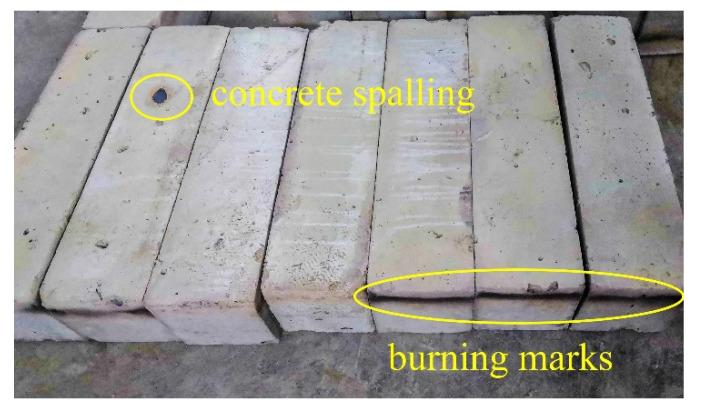
Apparent form of SFRC post-fire.

The specimens subjected to high temperatures by fire are shown in Figure 9. The fire-exposed surface turned to gray-white and became looser than that under the normal temperature conditions. On the fire-exposed surface of some specimens appeared some small cracks and a small amount of spalling. As in Figure 9, spalling was found on the specimen of steel fiber Type I with a dosage of 30 kg/m^3^. This was because the specimen had the lowest dosage, a shorter aspect ratio and a weaker adhesion than the other specimens. As no similar spalling was found on other specimens, it might be suggested that the mixing and stirring were not fully or evenly conducted when the steel fibers were added in the questioned specimen.

There are many evaluation methods for the flexural toughness of concrete members in the world, such as the ASTM C1018 standard [58] in the United States and the JSCE-SF4 standard [59] in Japan, but those standards have their limits. The ASTM C1018 standard [58] finds it difficult to accurately judge the position of the initial cracking point, which is greatly affected by human factors. The equivalent flexural strength in the JSCE-SF4 standard [59] is a dimensioned value, which is not easily used to analyze the specimens of different sizes. This paper adopted the CECS 13:2009 standard [51] of China Engineering Construction Standardization Association to evaluate the flexural toughness of SFRC. The CECS 13:2009 standard [51] has made an improvement compared with the ASTM C1018 standard [58] in that the flexural toughness of SFRC is evaluated by the equivalent flexural strength *f_e_*. This standard evaluation method is not affected by the position of the initial crack point. The initial crack point is the point at which the load–deflection curve changes from linear to nonlinear. For the deficiencies of the JSCE-SF4 standard [59], the CECS 13:2009 standard [51] proposes the flexural toughness ratio index *R_e_*, which is a dimensionless value, thus solving the problem in comparative analysis of specimens in different sizes. Therefore, the CECS 13:2009 standard [51] was used here to evaluate the flexural toughness of SFRC by the equivalent flexural strength *f_e_*, the flexural toughness ratio *R_e_* and the initial fracture strength *f_cr_* of fiber reinforced concrete (Formulas (3)–(5) [51]).

(3) $$f_{e}=\frac{\Omega_{k}L}{bh^{2}\delta_{k}}$$

(4) $$R_{e}=\frac{f_{e}}{f_{cr}}$$

(5) $$f_{cr}=\frac{F_{cr}L}{bh^{2}}$$

where *f_e_* is the equivalent flexural strength (MPa); *Ω_k_* is the area under load–deflection curve with mid-span deflection *L*/150; *L* is the span between test beam supports (mm); *b* is the specimen section width (mm); *h* is the specimen section height (mm); *δ_k_* is the deflection value of the mid-span deflection is *L*/150; *R_e_* is the flexural toughness ratio; *f_cr_* is the initial fracture strength of fiber-reinforced concrete (MPa); *F_cr_* is the initial crack flexural strength of fiber-reinforced concrete (N).

**Figure 10:**
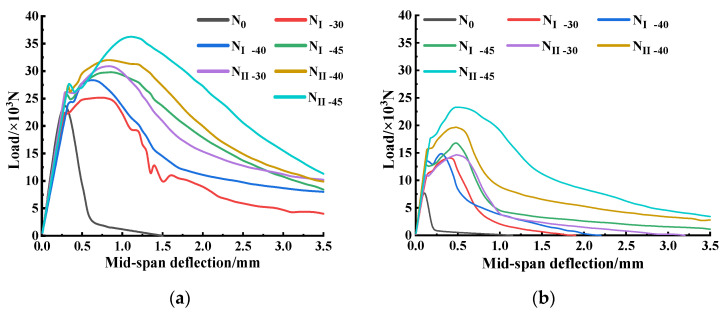
Load-deflection curve before and post-fire: (**a**) normal temperature conditions; (**b**) after-fire conditions.

The area under the *Ω_k_* curve was obtained using the origin software. The span *L* between the test supports was 300 mm. Figure 10 shows the load–deflection curves obtained from the four-point flexural test of different specimens before and after the high temperature. It is clear that steel fibers had a significant effect on the improvement of the equivalent flexural strength and the flexural toughness ratio of concrete at room temperature and high temperatures, but the envelope area of the load–deflection curve at room temperature was significantly larger than that after high temperature, indicating that the high temperature had a great influence on the equivalent flexural strength and flexural toughness ratio.

**Figure 11:**
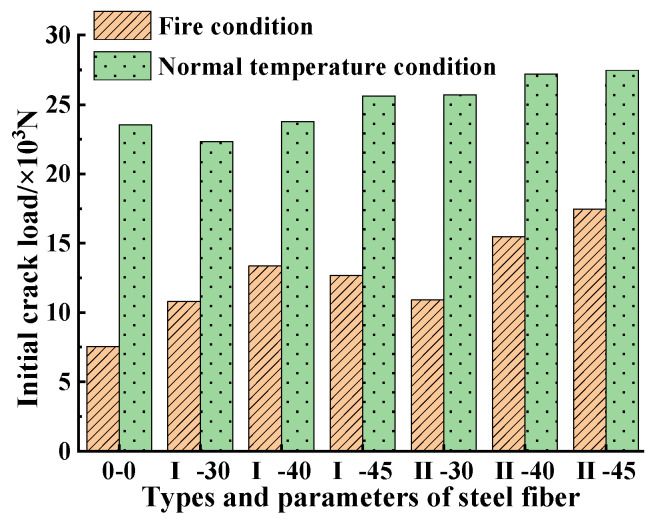
Initial crack flexural strength before and post fire.

It can also be seen from Figure 10 that the envelope area of the load-deflection curve of the SFRC Type II was larger than that of the SFRC Type I under the same dosage and the same working conditions, indicating that the addition of Type II had a greater ability to improve the flexural toughness than that of Type I, and a positive correlation was presented between steel fiber dosages and the flexural performance under both working conditions. When the steel fiber Type II dosage was 45 kg/m^3^, the SFRC had the best flexural performance. Figure 11 shows that under the same working conditions, the steel fiber dosage and type had little effect on the initial crack flexural strength (the load corresponding to the initial crack point), but the value of the initial crack flexural strength obviously decreased post-fire at a high temperature. The damage value to the initial crack flexural strength of the plain concrete reached 67.9%, higher than that of the steel fiber specimens, indicating that the addition of steel fibers helped reduce the effect of high temperature on the initial crack strength of concrete. (The damage value of the initial crack flexural strength is defined as: the difference between the initial crack load strength before and post-fire divided by the initial crack flexural strength at normal temperature).

**Figure 12:**
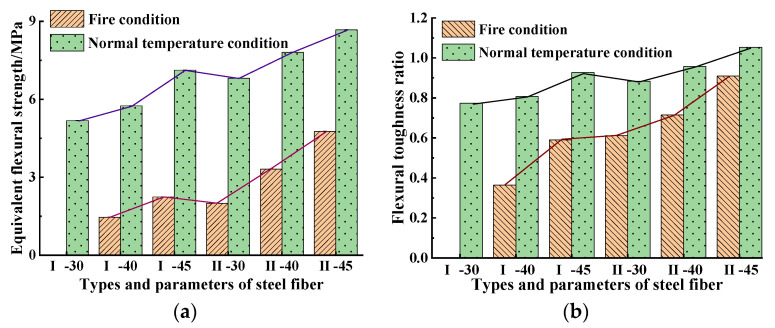
Comparison results of the equivalent flexural strength and flexural toughness ratio of specimens before and after high temperature: (**a**) equivalent flexural strength; (**b**) flexural toughness ratio.

According to the load–deflection curve, and Formula (3) to Formula (5) [51], the equivalent flexural strength and flexural toughness ratio of the specimens with different steel fiber types and dosages were obtained, respectively. The calculation results are shown in Table 7 and Table 8 and Figure 12.

Figure 12 shows that under both working conditions the ratio of equivalent flexural strength to flexural toughness of SFRC all increased with the increase in steel fiber dosages. The damage value of the equivalent flexural strength of SFRC Type I in dosage of 30 kg/m^3^ was almost 100%. Compared with other steel fiber types and dosages, the minimum damage of Type II at a dosage of 45 kg/m^3^ was 45.2%. The equivalent flexural strength of Type I at a dosage of 45 kg/m^3^ was 1.5 times that of Type I at a dosage of 40 kg/m^3^; the equivalent flexural strength of Type II at a dosage of 45 kg/m^3^ was 1.4 and 2.4 times that of Type II in dosages of 40 kg/m^3^ and 30 kg/m^3^, respectively. At the same dosage, the equivalent flexural strength of Type II was higher than that of Type I, among which, that of the steel fiber specimen at a dosage of 45 kg/m^3^ increased by 52.9%. The maximum equivalent flexural strength of Type II at a dosage of 45 kg/m^3^ was 8.68 MPa, which was 1.1 and 1.3 times that in 40 kg/m^3^ and 30 kg/m^3^, increasing by 17.8% compared with SFRC Type I in the same dosage.

Under both working conditions, the flexural toughness ratio of SFRC reached the maximum value when steel fiber Type II in dosage of 45 kg/m^3^ was adopted, and the improving of the flexural toughness ratio of steel fiber Type I was significantly less than that of Type II. Under high temperature conditions, the flexural toughness ratio of Type II in dosage of 45 kg/m^3^ was 1.5 times that of Type I in the same dosage, only reduced by 13.3% compared with that under normal temperature conditions. That damage reduction value was the minimum damage value of the flexural toughness ratio among all steel fiber dosages and types.

## 4. Thermal Conductivity Study of SFRC Based on ISO 834 Standard Fire Test

### 4.1. Basic Assumptions

According to the principles of heat transfer, there are three basic ways of heat transfer: heat conduction, heat convection and heat radiation [60]. The thermal parameters involved in the heat transfer process of SFRC are mainly thermal conductivity, specific heat, convective heat transfer coefficient, comprehensive radiation coefficient and so on. To simplify the analysis process of the numerical calculation model of the SFRC temperature field, the following assumptions were made:

(1) the temperature field analysis involved here was an uncoupled heat transfer analysis, so the stress and strain had no effect on the temperature field of the model;
(2) the heat released by the SFRC specimens during the heating process due to a series of internal chemical reactions was ignored;
(3) the heat loss due to the evaporation of pore water inside the SFRC specimens during the heating process was not considered;
(4) it was assumed that the concrete and steel fibers were isotropic, and all isotropic thermal parameters were the same value;
(5) except for the surface that was in direct contact with the open flame, the rest sides of the specimens werethermal insulation surfaces.

### 4.2. Numerical Modeling

#### 4.2.1. Thermal Parameter Selection

In calculating the temperature field of concrete components, the heat conduction is a nonlinear transient issue because the thermal conductivity and specific heat of concrete are not constant. The concrete heat conduction differential formula is a nonlinear parabolic partial differential formula. The basic differential formula for the transient heat conduction of concrete is Formula (6) [61]:

(6) $$\rho C\frac{\partial T}{\partial t}=\frac{\partial }{\partial x}\left(\lambda \frac{\partial T}{\partial x}\right)+\frac{\partial }{\partial y}\left(\lambda \frac{\partial T}{\partial y}\right)+\frac{\partial }{\partial z}\left(\lambda \frac{\partial T}{\partial z}\right)+g$$

where *ρ* is the material density (kg/m^3^); *C* is the specific heat of the material (J/(kg·K)); *λ* is the thermal conductivity of the material (W/(m·K)); *T* is the temperature of the material at any time (°C); *g* is the heat generated per unit volume of the material in per unit time (W/m^3^).

It is generally agreed that in the calculation of the temperature field of the concrete beam, the temperature along the longitudinal direction of the concrete beam is the same, and there is no heat source inside the component, that is, *g* is taken as 0. Therefore, the three-dimensional temperature field issue of the above formula can be simplified to a two-dimensional temperature field issue along the section [62], and the concrete heat conduction formula can be abbreviated as Formula (7) [63]:

(7) $$\rho_{c}C_{c}\frac{\partial T}{\partial t}=\frac{\partial }{\partial x}\left(\lambda_{c}\frac{\partial T}{\partial x}\right)+\frac{\partial }{\partial y}\left(\lambda_{c}\frac{\partial T}{\partial y}\right)$$

where *ρ*_c_ is the density of concrete (kg/m^3^); *C*_c_ is the specific heat of concrete (J/(kg·K)); *λ*_c_ is the thermal conductivity of concrete (W/(m·K)).

The main thermal parameters involved in the above Formula (7) are: *λ*_c_, *C*_c_, and *ρ*_c_, among which, *λ*_c_ varies greatly due to different concrete types, and is believed in negative correlation with temperature *T* [64]. The existing literature has different ranges and methods of thermal parameters [62,65,66]. Currently, the parameter values in the Eurocode for the design of concrete structures are generally accepted [35,67]. The upper limit of Formula (8) of the thermal conductivity is obtained in the tests of steel and concrete composite structures, and the lower limit of Formula (9) is obtained in the tests of ordinary concrete structures [35,67]. So, here in the numerical simulation of the temperature field, the upper limit values of *λ*_c_ in the Eurocode were referred to for calculation, which is Formula (8), and *C*_c_ as to Formula (10). The thermal conductivity *λ*_s_ and specific heat *C*_s_ of steel fibers were the same as those of ordinary steel. Referring to the calculation formula of the European Code for Design of Steel Structures [68], the calculation formulas of *λ*_s_ and *C*_s_ were (11) and (12), respectively.

The upper limit of the thermal conductivity of concrete:

(8) $$\lambda_{c}=2-0.2451(T/100)+0.0107{\left(T/100\right)}^{2}\;(20\;{}^{\circ}C\leq T\leq 1200\;{}^{\circ}C)$$

The lower limit of the thermal conductivity of concrete:

(9) $$\lambda_{c}=1.36-0.136(T/100)+0.0057{\left(T/100\right)}^{2}\;(20\;{}^{\circ}C\leq T\leq 1200\;{}^{\circ}C)$$

The specific heat of concrete:

(10) $$C_{c}=\left\{\begin{array}{l} 900\;\;\;\;\;\;\;\;(20\;{}^{\circ}C\leq T\leq 100\;{}^{\circ}C)\\ 900+(T-100)\;\;\;(100\;{}^{\circ}C\leq T\leq 200\;{}^{\circ}C)\\ 900+(T-200)/2\;\;(200\;{}^{\circ}C\leq T\leq 400\;{}^{\circ}C)\\ 1100\;\;\;\;\;\;\;(400\;{}^{\circ}C\leq T\leq 1200\;{}^{\circ}C) \end{array}\right.$$

The thermal conductivity of steel fiber:

(11) $$\lambda_{s}=\left\{\begin{array}{l} 54-3.33\times 10^{-2}\times T\;\;(20\;{}^{\circ}C\leq T\leq 800\;{}^{\circ}C)\\ 27.3\;\;\;\;\;\;\;\;(800\;{}^{\circ}C\leq T\leq 1200\;{}^{\circ}C) \end{array}\right.$$

The specific heat of steel fiber:

(12) $$C_{s}=\left\{\begin{array}{l} 425+7.73\times 10^{-1}\times T-1.69\times 10^{-3}\times T^{2}+2.22\times 10^{-6}\times T^{3}\;(20\;{}^{\circ}C\leq T\leq 600\;{}^{\circ}C)\\ 666+13002/(738-T)\;\;\;\;\;\;\;\;\;\;\;\;\;\;\;\;(600\;{}^{\circ}C\leq T\leq 735\;{}^{\circ}C)\\ 545+17820/(T-731)\;\;\;\;\;\;\;\;\;\;\;\;\;\;\;\;(735\;{}^{\circ}C\leq T\leq 900\;{}^{\circ}C)\\ 650\;\;\;\;\;\;\;\;\;\;\;\;\;\;\;\;\;\;\;\;\;\;\;(900\;{}^{\circ}C\leq T\leq 1200\;{}^{\circ}C) \end{array}\right.$$

where *C*_s_ is specific heat for steel fibers (J/(kg·K)); *λ*_s_ is the thermal conductivity of steel fibers (W/(m·K)); *T* is the temperature of the material at any time (°C).

#### 4.2.2. Parameter Settings and Boundary Conditions

**Figure 13:**
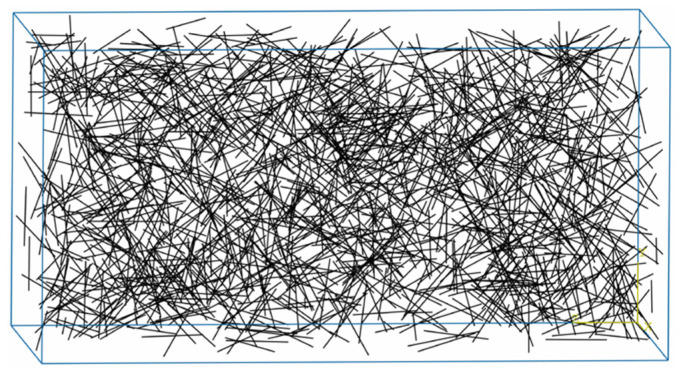
Perspective view of the SFRC model.

Concrete and steel fibers were set up independently in the ABAQUS model, and their material properties of concrete and steel fibers needed to be investigated separately. The concrete size was established according to the test prototype, which was 150 × 150 × 300 mm. The concrete was established according to the test prototype, its thermal conductivity and specific heat were calculated according to Formulas (8) and (10), and concrete density took a constant value of 2400 kg/m^3^. The steel fiber was established with the optimum dosage of 45 kg/m^3^ of Type II, its thermal conductivity and specific heat were calculated according to Formulas (11) and (12), and the steel fiber density was set at 7800 kg/m^3^. It is worth noting that the steel fiber model was established without considering the geometry. In other words, straight and flat steel fibers with a circular cross-section were used in the numerical simulation. According to the amount of steel fibers, 1417 fibers were generated and assembled randomly and uniformly in the concrete model, as shown in Figure 13.

In the numerical analysis, the analysis step was set to transient heat transfer, the duration time of single-side fire was 60 min and the ambient temperature at the start of the modeling was the same as the temperature in the furnace before the test, which was 15 °C. Except the fire surface, the other surfaces of SFRC specimens were covered with refractory cotton for thermal insulation, so they were regarded as thermal insulation surfaces. The absolute zero degree was fixed as −273 °C, and the Stefan-Boltzmann constant was 5.67 × 10^−8^ W/(m^2^·K^4^) [69], which were used to simulate boundary conditions similar to those of the SFRC specimens in the test. According to Eurocode [44], the convective heat exchange coefficient of the fire surface was set as 25 W/(m·K), the non-fired surface was regarded as the radiative convective heat exchange boundary, and the convective heat exchange coefficient was taken as 9 W/(m·K). The concrete grid was set to DC3D8 three-dimensional eight-node thermal element, and the side length of the grid cell was 5 mm; the steel fiber grid was set to DC1D2 one-dimensional two-node thermal element, and the side length of the grid cell was 7 mm. The temperature field calculation was thus finally carried.

### 4.3. Temperature Field Calculation Results and Analysis

**Figure 14:**
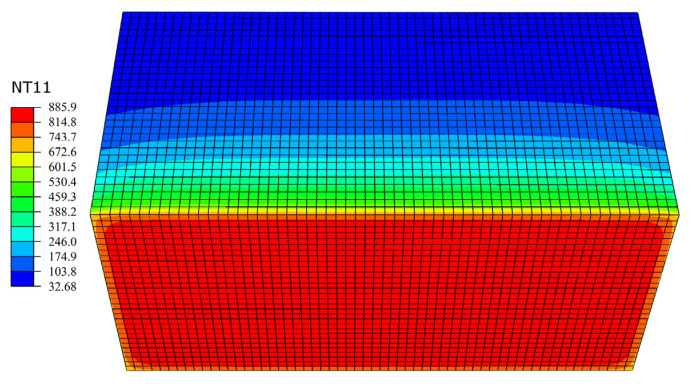
Temperature field cloud diagram of the SFRC specimen (°C).

**Figure 15:**
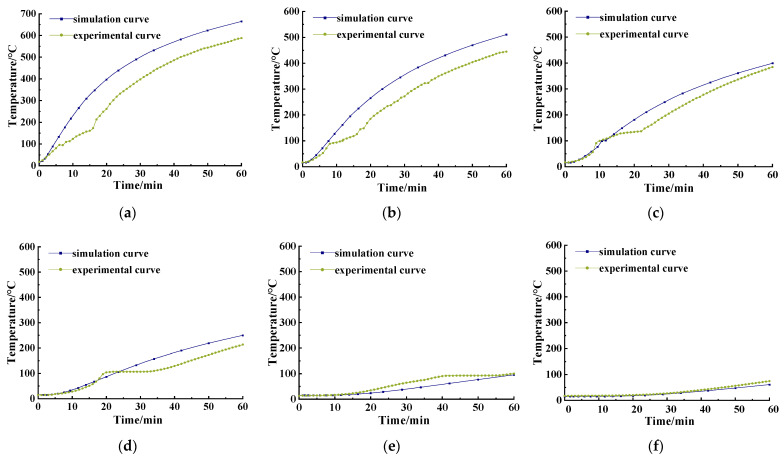
Temperature–time curve comparison at each section based on *λ*_c_ simulation and experiment: (**a**) 10 mm; (**b**) 20 mm; (**c**) 30 mm; (**d**) 50 mm; (**e**) 80 mm; (**f**) 120 mm.

The cloud diagram of the calculated temperature field is shown in Figure 14. It can be seen that the temperature field was distributed in a gradient along the longitudinal direction of the specimen and the color shade of the temperature field is negatively correlated with the distance from the fire surface. According to the calculation results of numerical simulation, the temperature changed with the test time at six different depths of 10, 20, 30, 50, 80 and 120 mm from the fire surface are obtained from the model, and so were the temperature–time curves measured at the corresponding position. The comparison is shown in Figure 15.

It can be seen from Figure 15 that when the temperature did not exceed 100 °C, the simulation results and the test results were relatively close to each other at 10, 20, 30, 50, 80, and 120 mm from the fire surface. As a 60 min heating time was adopted in the test, and the temperature inside the furnace was limited, SFRC could not exert its thermal conductivity well. Therefore, the temperature rose to 100 °C at 80 mm from the fire surface, and that at 120 mm did not reach 100 °C. When the temperature exceeded 100 °C, a temperature plateau appeared due to the evaporation and heat absorption of the moisture inside the concrete. So, the test curve had a hysteresis compared with the simulation curve at 10, 20, 30 and 50 mm, but the test curve seemed consistent with the overall change trend of the simulated curve. To obtain a more reasonable simulation result, an inversion study of the thermal parameters of the materials was conducted based on the test data.

### 4.4. Proposition of SFRC Thermal Conductivity

Upon the comparison of the temperature field between the above test values and the simulated values, it was clear that there was a certain deviation between the temperature conditions calculated by using the upper limit of the thermal parameters of the Eurocode and the test values. That upper limit was too conservative. The research of Kodur et al. [32] showed that temperature had little effect on the specific heat of steel fibers; Nagy et al. [70] proposed that the change of thermal conductivity of steel fibers was mainly related to the density of steel fibers; if ignoring the pore water inside SFRC components, the effect of temperature on the specific heat of concrete was smaller than the thermal conductivity [67]. Therefore, under the condition that the thermal parameters of steel fiber remained unchanged and the specific heat *C*_c_ and density *ρ* of concrete remained unchanged, the *λ*_SFRC_ Formula (13) was put forward here based on Formula (8), so that the temperature–time curve calculated by the simulation was more consistent with the test values.

(13) $$\lambda_{\mathrm{SFRC}}=1.5-0.19(T/100)+0.005{\left(T/100\right)}^{2}\;(20\;{}^{\circ}C\leq T\leq 1200\;{}^{\circ}C)$$

**Figure 16:**
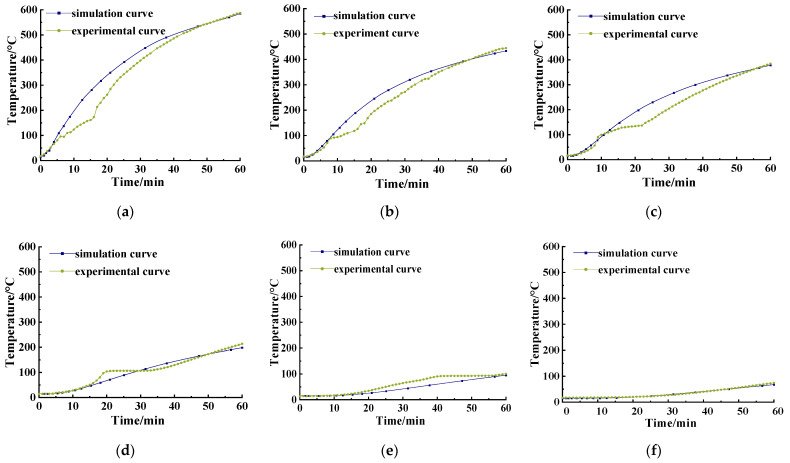
Temperature–time comparison curves at each section based on the *λ*_SFRC_ simulation and experiment: (**a**) 10 mm; (**b**) 20 mm; (**c**) 30 mm; (**d**) 50 mm; (**e**) 80 mm; (**f**) 120 mm.

According to Formula (13), the SFRC temperature field was calculated, verified and compared with the test values, the temperature–time curves at different distances from the fire surface thus obtained are shown in Figure 16. Table 9 shows the temperature comparison between the test values of each section and the simulated values calculated by *λ*_c_ and *λ*_SFRC_ when exposed to fire for 60 min.

Comparing Figure 15 and Figure 16, it is clear that the temperature–time curve of the simulated value based on *λ*_SFRC_ was closer to the test values, and the temperature difference from the test data when exposed to fire for 60 min changes greatly compared with the *λ*_c_ simulation. Combining with Table 9, it can be said that at a distance of 10 mm from the fire surface, the temperature difference between the front and rear simulated values and the test values decreased from 77.24 °C to 2.47 °C and decreased by 74.77 °C. This indicates that the temperature field calculated by *λ*_SFRC_ was closer to the test values, which proves that *λ*_SFRC_ is more suitable for the calculation of the temperature field of SFRC structure.

## 5. Conclusions

Based on the ISO 834 standard heating curve, an open flame test was carried out on SFRC, and the distribution law of the internal temperature field of the SFRC structure under single-side fire was studied. Through the tests on the standard flexural toughness of the concrete specimens in different steel fiber types and dosages, the damage degree of equivalent flexural strength and flexural toughness of SFRC was ascertained compared with plain concrete. The temperature field of SFRC under standard fire environment was simulated and analyzed by using ABAQUS numerical simulation software, and a calculation formula of thermal conductivity of SFRC was proposed. The main conclusions are as follows:

(1) Incorporation of steel fibers into concrete helps decrease the temperature gradient inside the concrete, reduce the internal thermal stress difference and improve the crack resistance and fire resistance of the concrete;
(2) Compared with each SFRC specimen, the plain concrete specimen had a higher damage value of the initial crack flexural strength, which indicates that the addition of steel fibers helps reduce the high temperature impact on the degree of damage to the initial crack flexural strength of concrete;
(3) Steel fibers can significantly improve the equivalent flexural strength of concrete under both normal and high temperature conditions. Moreover, in the scope of this study, the equivalent flexural strength increased with the increase in steel fiber dosages;
(4) The simulated temperature value of λ_SFRC_ was more similar to the test values than that of λ_c_, which verifies the rationality of formula λ_SFRC_.

## Figures and Tables

**Table 1:** Steel fiber parameters.

Steel Fiber Form	Length (mm)	Diameter (mm)	Slenderness Ratio	Tensile Strength (MPa)	Elastic Modulus (GPa)	Density (kg/m^3^)	Limiting Drawing Ratio (%)
I	25	0.50	50	950	200	7800	0.5–3.5
II	35	0.50	70	950	200	7800	0.5–3.5

**Table 2:** C35 concrete mix proportions.

Cement (kg/m^3^)	River Sand (kg/m^3^)	Gravel (kg/m^3^)	Water (kg/m^3^)	Water-Cement Ratio	Percentage of Sand (%)
370	672	1200	185	0.5	36

**Table 3:** Grouping and numbering of specimens under normal temperature conditions.

Specimen Number	N_I-30_	N_I-40_	N_I-45_	N_II-30_	N_II-40_	N_II-__45_	N_0_
Steel fiber dosage (kg/m^3^)	30	40	45	30	40	45	-
Slenderness ratio	50	50	50	70	70	70	-
Quantity	3	3	3	3	3	3	3

**Table 4:** Grouping and numbering of specimens under the fire condition.

Specimen Number	F_I-30_	F_I-40_	F_I-45_	F_II-30_	F_II-40_	F_II-45_	F_0_
Steel fiber dosage (kg/m^3^)	30	40	45	30	40	45	-
Slenderness ratio	50	50	50	70	70	70	-
Quantity	3	3	3	3	3	3	3

**Table 5:** Grouping and numbering of embedded thermocouple specimens.

Specimen Number	B_0_	B_II-30_	B_II-40_	B_II-45_
Steel fiber dosage (kg/m^3^)	-	30	40	45
Slenderness ratio	-	70	70	70
Quantity	1	1	1	1
Sensor quantity	6	6	6	6

**Table 6:** Temperature values (°C) in different dosages and from different fire exposure distances at 60 min heating time.

Distances	TB_II-45_	TB_II-40_	TB_II-30_	TB_II-0_
10 mm	587.6	534.8	502.6	464.9
20 mm	444.7	428.1	421.1	395.2
30 mm	384.5	377.7	351.4	320.2
50 mm	213.3	197.3	193.7	161.9

**Table 7:** Calculation results of the mechanical properties of SFRC before fire.

Specimens	N_0_	N_I-30_	N_I-40_	N_I-45_	N_II-30_	N_II-40_	N_II-45_
*F_cr_*/kN	23.53	22.33	23.77	25.63	25.7	27.2	27.47
*f_cr_*/MPa	7.06	6.7	7.13	7.69	7.71	8.16	8.24
*Ω_k_*/kN·mm	/	34.54	38.33	47.50	45.42	52.06	57.83
δ*_k_*/mm	/	2	2	2	2	2	2
*f_e_*/MPa	/	5.18	5.75	7.13	6.81	7.81	8.68
*R_e_*	/	0.77	0.81	0.93	0.88	0.96	1.05

**Table 8:** Calculation results of the mechanical properties of SFRC after fire.

Specimens	F_0_	F_I-30_	F_I-40_	F_I-45_	F_II-30_	F_II-40_	F_II-45_
*F_cr_*/kN	7.54	10.79	13.37	12.67	10.90	15.47	17.47
*f_cr_*/MPa	2.26	3.24	4.01	3.80	3.27	4.64	5.24
*Ω_k_*/kN·mm	/	/	9.75	14.95	13.33	22.11	31.76
δ*_k_*/mm	/	/	2	2	2	2	2
*f_e_*/MPa	/	/	1.46	2.24	2.00	3.32	4.76
*R_e_*	/	/	0.36	0.59	0.61	0.71	0.91

**Table 9:** Temperature comparison of each section based on different calculation formulas.

Distance from fire surface		10 mm	20 mm	30 mm	50 mm	80 mm	120 mm
	Test value (°C)	587.60	444.70	384.48	213.33	100.40	74.36
Simulation based on *λ*_c_	Simulation value (°C)	664.84	510.80	399.06	249.99	95.47	60.87
	Temperature difference (°C)	77.24	66.10	14.58	36.66	4.93	13.49
Simulation based on *λ*_SFRC_	Simulation value (°C)	585.13	433.52	377.99	198.26	94.96	67.33
	Temperature difference (°C)	2.47	11.18	6.49	15.07	5.44	10.03

## Data Availability

Not applicable.
